# Endometriosis-related infertility in China: analysis of the global burden of disease study 2021

**DOI:** 10.3389/fpubh.2025.1651254

**Published:** 2025-10-07

**Authors:** Jianting Lao, Dongyi Shen, Panwei Hu, Yanhua Song, Hong Yang

**Affiliations:** ^1^Department of Gynaecology, Shanghai Municipal Hospital of Traditional Chinese Medicine, Shanghai University of Traditional Chinese Medicine, Shanghai, China; ^2^Department of Gynaecology and Obstetrics, Shuguang Hospital Affiliated to Shanghai University of Traditional Chinese Medicine, Shanghai, China

**Keywords:** global burden of disease, endometriosis, female infertility, prevalence, age-period-cohort, China

## Abstract

**Objective:**

Utilizing the Global Burden of Disease (GBD) 2021 dataset, this study analyzed temporal trends (1990–2021) and age-period-cohort patterns of endometriosis-related infertility burden in China versus globally. By employing joinpoint regression and APC modeling, we aimed to identify key epidemiological shifts and disparities, thereby providing evidence-based insights for optimizing health service planning and resource allocation strategies targeting endometriosis-related infertility.

**Methods:**

Using age-standardized prevalence rates (ASPR) of endometriosis-related infertility (1990–2021) from the GBD 2021 database, we analyzed Chinese females aged 15–49 years. Joinpoint regression identified significant trend changes in ASPR, while age-period-cohort (APC) modeling decomposed effects into age, period, and birth cohort dimensions using 5-year intervals.

**Results:**

In 2021, the age-standardized prevalence rate (ASPR) of patients with endometriosis-related primary infertility in China was 280.63 per 100,000 population (95% UI: 115.61 to 616.39). The percentage change in ASPR for endometriosis-associated primary infertility in China between 1990 and 2021 was −23.35% (95% UI: −10.52% to −32.68%) above the global level −29.61% (95% UI: −37.19% to −24.79%). In 2021, the ASPR of patients with endometriosis-associated secondary infertility in China was 1849.35 per 100,000 population (95% UI: 1104.61 to 2931.00). The percentage change of endometriosis-associated secondary between 1990 and 2021 ASPR in China was −38.16% (95% UI: −43.81% to −31.53%), below the global level −24.70% (95% UI: −27.99% to −21.11%).

**Conclusion:**

The ASPR of endometriosis-related female infertility in China and globally declined between 1990 and 2021. This indicates that China has attained significant progress in addressing and managing endometriosis-related female infertility. However, the overall burden of endometriosis-related female infertility remains substantial and requires continued attention.

## Introduction

Endometriosis is an estrogen-dependent gynecological disorder characterized by the presence of endometrial glands and stromal tissue outside the uterus (1). Endometriosis affects approximately 10% of girls and women of reproductive age globally and is associated with a significant impact on fertility, overall well-being, and healthcare expenses (2). Compared to the general population, individuals with endometriosis face an approximately two-fold to four-fold increased risk of infertility, while the likelihood of infertile patients developing endometriosis is up to approximately 50% (3).

Although endometriosis itself may not be the primary cause of infertility (4), it contributes to infertility in affected women through factors such as pain, inflammation, changes in pelvic anatomy and adhesions, ovarian dysfunction, and decreased endometrial receptivity (5).

Female infertility comprises two main categories: primary infertility, where a woman has not become pregnant despite not using contraception with the same partner for at least 12 months, and secondary infertility, where a woman has not become pregnant for at least 12 months since her last pregnancy, despite not using contraception with the same sexual partner (6, 7). Approximately 17.8% of individuals in high-income countries and 16.5% in low- or middle-income countries face challenges with fertility (8). China, as the world’s most populous nation, faces unique demographic pressures: rapid fertility decline and accelerated population aging. Over the past 30 years, Chinese women have been delaying their first marriage while also experiencing a significant decline in average birth rates (9). From 1990 to 2019, the number of female infertility cases in China increased by 7.06 million, while the number of cases worldwide grew by 56.71 million. The average annual growth rates of age-standardized prevalence rate (ASPR) were 10.10 and 7.28%, respectively (10). Infertility can negatively impact a couple’s mental health and sexual relationships, leading to reduced quality of life (11). In addition, declining birth rates may exacerbate issues related to an aging population.

The previous GBD 2019 study conducted an in-depth assessment of the disease burden attributed to polycystic ovarian syndrome in China (12). In the present study, we used the latest data from GBD 2021 (13). The ASPR of endometriosis-related infertility burden in China was examined, focusing on both primary and secondary infertility, with comparisons to global levels. In addition, this study assessed the impact of endometriosis-related infertility in China using joinpoint regression and age-period-cohort (APC) analyses. Employing the latest extensive population health statistics, this study presented findings on the impact of endometriosis-related infertility in China, highlighting the significance of understanding epidemiological patterns to inform healthcare and community wellness strategies. Given the complexity of infertility pathogenesis, it is critical to continue research on the causes and development of treatment strategies. Our research seeks to offer fundamental insights for governmental bodies and healthcare policymakers, facilitate more efficient decision-making and judicious resource allocation, and provide a scientific basis for health service planning for endometriosis-related infertility.

## Methods

### Study population and setting

This analysis utilized modeled epidemiological data from the Global Burden of Disease Study 2021 (GBD 2021), representing females aged 20–49 years in China (1990–2021). As GBD synthesizes data sources (e.g., surveys, registries, literature) using standardized modeling, no primary participant recruitment occurred. Population estimates were derived from nationally representative censuses and demographic surveillance systems. Findings reflect population-level trends applicable to resource allocation and policy planning across China.

### Data sources

Endometriosis-related infertility data for the period 1990–2021 were obtained from the Global Health Data Exchange query tool.^1^ The GBD periodically revises projections of hazard exposure rates, comparative health risks of exposure, and the fraction of disease impact linked to specific ailments or injuries attributed to particular risk factors, generally categorized into environmental and occupational, lifestyle, and physiological risk clusters (14). GBD 2021, the most recent data published in the database, provides extensive evaluations of exposure rates, comparative health hazards, and associated disease impacts for 88 risk factors across 204 nations and territories, as well as 811 locations, spanning from 1990 to 2021 (14). According to the GBD 2021 study, endometriosis cases were defined in accordance with the American College of Obstetricians and Gynecologists (ACOG) guidelines, requiring diagnosis through pelvic examination confirmed via laparoscopy or laparotomy. Under the International Classification of Diseases, Tenth Revision (ICD-10), endometriosis is coded under N80 to N80.9, while infertility is classified under N97 to N97.9 (15).

The GBD 2021 meets the recommendations of the Guidelines for Accurate and Transparent Health Estimates Reporting (GATHER) (16). Because the data were obtained from a publicly available database, the need for ethical approval was waived.

### Descriptive analysis

The primary outcome measure in our study was the ASPR. Disease prevalence is defined as the proportion of individuals within a population who have the condition at a specific point in time, expressed per thousand and rates per 100,000 population. The GBD World Population Age Standard was employed to compute the age-standardized population. All estimates were generated with a 95% uncertainty interval (95% UI), determined from the 2.5th and 97.5th sorting percentiles of 1,000 plots from the uncertainty distribution. Statistical analysis was conducted using R version 4.3.1 (R Foundation, Vienna, Austria).

### Joinpoint regression analysis

Connection-point regression models are sets of linear statistical models used to evaluate trends in the disease burden of endometriosis-associated infertility over time. These models estimate changes in disease rates using the least-squares method, thereby mitigating the subjectivity inherent in a typical linear trend analysis. The turning point of the trend was identified by minimizing the squared deviations between observed and predicted values using a grid search technique. The significance of the observed changes was assessed using the Monte Carlo permutation method. The average annual percentage change (AAPC) was calculated to estimate the overall trend in the burden of endometriosis-related infertility from 1990 to 2021. AAPC > 0 signifies an ascending trend, whereas AAPC < 0 indicates a descending trend. Joinpoint software (version 4.9.1.0; National Cancer Institute, Rockville, MD, USA) was employed to establish this model, with *p* < 0.05 considered statistically significant.

### APC analysis model

We utilized an age-period-cohort (APC) model to analyze age-related biological progression, contemporary medical and social influences (period effects), and early-life exposures (cohort effects) on endometriosis-related infertility prevalence. The model generated three key outputs: (1) Net drift (overall annual % prevalence change, combining period and cohort effects), where values >0 indicate rising burden and <0 declining burden; (2) Local drift (age-specific annual % change), identifying high-risk age groups; and (3) Rate ratios (RR) for period/cohort (relative risk vs. reference), with RR > 1 signaling elevated risk and RR < 1 reduced risk. To avoid cohort overlap, age/period/cohort intervals were fixed at 5 years. The reference period was 2002–2006 (RR = 1) and reference birth cohort was 1982–1991 (RR = 1). Statistical significance (*p* < 0.05) was assessed via longitudinal age curves and RR confidence intervals (17).

## Results

### The burden of endometriosis-associated infertility in China and globally between 1990 and 2021

**Figure 1:**
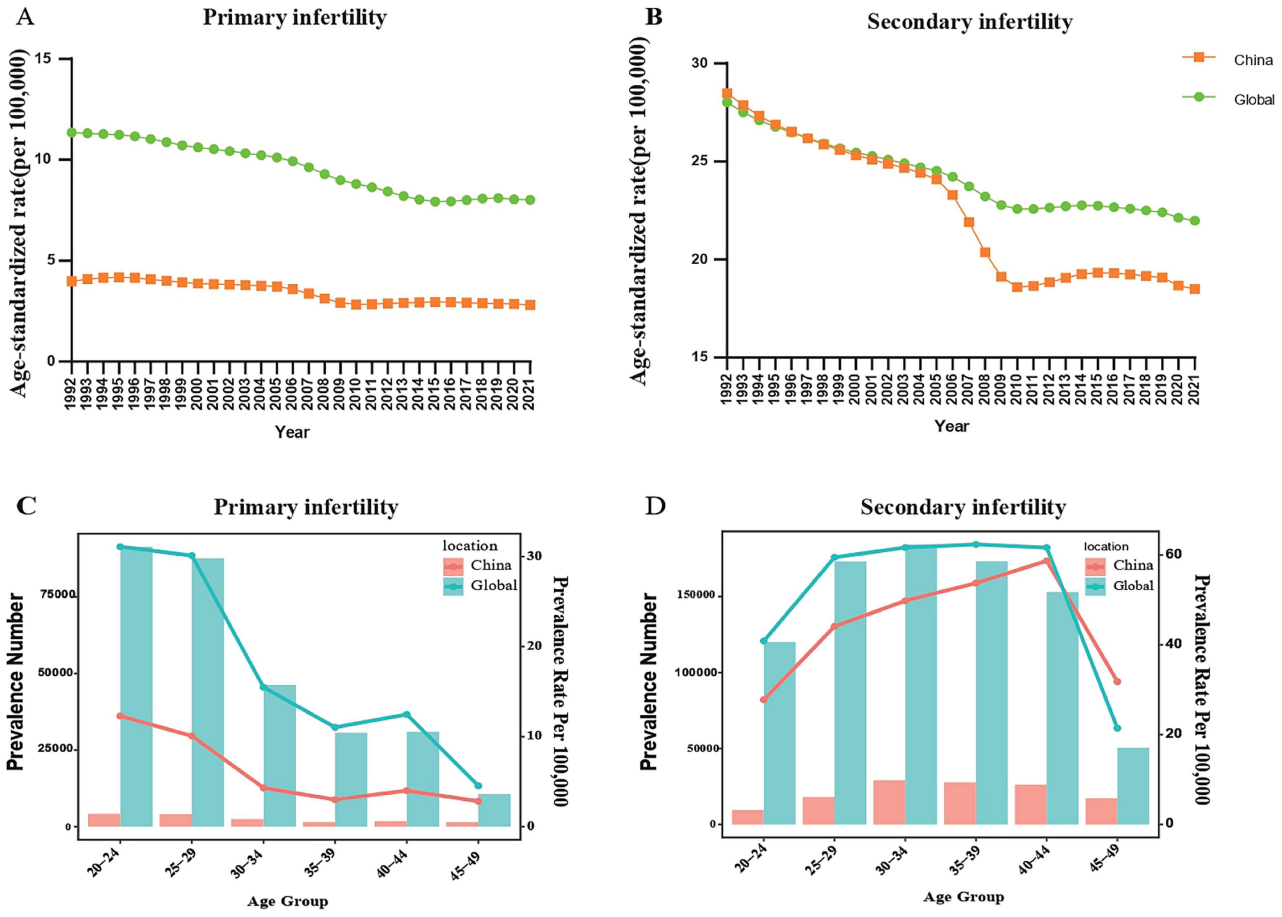
**(A)** Trend for age-standardized prevalence rate (ASPR) of endometriosis-related primary infertility in China and globally from 1990 to 2021. **(B)** The trend for ASPR of endometriosis-related primary infertility in China and globally from 1990 to 2021. **(C)** Prevalence cases and prevalence rates of endometriosis-related primary infertility by age group in China and globally in 2021. **(D)** Prevalence cases and prevalence rate of endometriosis-related secondary infertility by age group in China and globally in 2021.

In 2021, the global prevalence of endometriosis-related primary infertility across ages was 312.36 thousand cases (95% UI: 155.32 to 585.83), with ASPR of 802.02 per 100,000 population (95% UI: 396.98 to 1518.31). The percentage change in ASPR for endometriosis-associated primary infertility globally between 1990 and 2021 was −29.61% (95% UI: −37.19% to −24.79%) (Supplementary Table 1; Figure 1A).

In 2021, the number of patients with endometriosis-associated primary infertility of all ages in China was 16.40 thousand cases (95% UI: 7.01 to 33.40), with ASPR of 280.63 per 100,000 population (95% UI: 115.61–616.39). The percentage change of ASPR for endometriosis-associated primary infertility in China between 1990 and 2021 was −23.35% (95% UI: −10.52% to −32.68%) (Supplementary Table 1; Figure 1A).

In 2021, the global prevalence owing to endometriosis-associated secondary infertility across all ages was 873.54 thousand cases (95% UI: 504.75 to 1445.04), with ASPR of 2197.65 per 100,000 population (95% UI: 1269.91 to 3630.90). The percentage change in ASPR for endometriosis-related secondary infertility globally between 1990 and 2021 was −24.70% (95% UI: −27.99% to −21.11%) (Supplementary Table 1; Figure 1B).

In 2021, the number of patients with endometriosis-related secondary infertility of all ages in China was 129.11 thousand cases (95% UI: 77.07 to 208.23), with ASPR of 1849.35 per 100,000 population (95% UI: 1104.61 to 2931.00). The percentage change in ASPR for endometriosis-related secondary infertility in China between 1990 and 2021 was −38.16% (95% UI: −43.81% to −31.53%) (Supplementary Table 1; Figure 1B).

### Epidemic situation of endometriosis-related infertility among different age groups in China and globally in 2021

In 2021, the influence of age on endometriosis-related primary infertility in China and worldwide showed a decreasing trend between the ages of 20 and 49. In the 20–24 age group, the number of endometriosis-related primary infertility cases was highest, reaching 4.22 thousand cases in China (95% UI: 0.95 to 11.45) and 91.32 thousand cases globally (95% UI: 31.19 to 197.86) (Supplementary Table 1; Figure 1C). Similarly, the prevalence of endometriosis-related primary infertility was highest in the 20–24 age group. The prevalence rate in China was 12.30 per 100,000 population (95% UI: 2.76 to 33.37), while globally it was 31.09 per population (95% UI: 10.62 to 67.36) (Supplementary Table 1; Figure 1C).

In 2021, the influence of age on endometriosis-related secondary infertility in both China and the world exhibited a trend of initial rise followed by a decline between the ages of 20 and 49 years. The number of endometriosis-related secondary infertility cases was highest in the 30–34 age group, reaching 29.12 thousand cases in China (95% UI: 15.34 to 49.31) and 184.33 thousand cases globally (95% UI: 91.35 to 321.89) (Supplementary Table 1; Figure 1D). Similarly, in 2021, the prevalence of endometriosis-related secondary infertility in both China and the world exhibited an initial rise followed by a decline. The key difference is that in China, the prevalence rate of endometriosis-related secondary infertility shows an increase up to the 40–44 age group, reaching a peak of 58.70 per 100,000 population (95% UI: 31.43 to 99.04) in this age group. In contrast, the global prevalence remained relatively stable between the ages of 25–29 and 40–44, reaching a peak of 62.37 per 100,000 population (95% UI: 33.78 to 103.30) (Supplementary Table 1; Figure 1D).

### Joinpoint regression analysis

**Figure 2:**
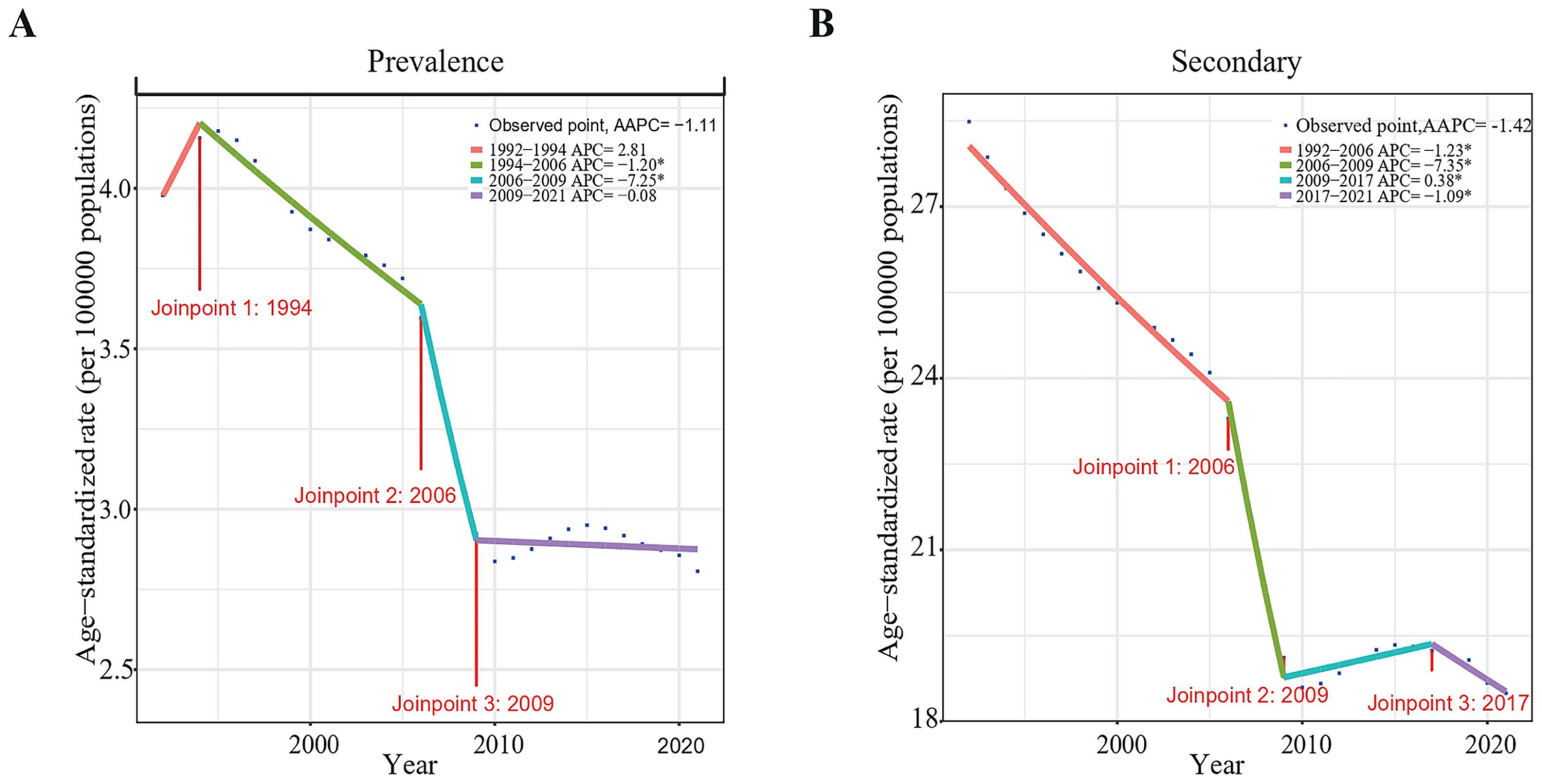
Joinpoint regression analysis of the ASPR of endometriosis-related infertility in China from 1990 to 2021. **(A)** Primary infertility. **(B)** Secondary infertility.

Joinpoint regression analysis determined that the age-standardized rates of endometriosis-related infertility burden indicators, including AAPC and annual percentage change (APC) showed a declining trend in China. From 1990 to 2021, the AAPC for ASPR of primary female infertility related to endometriosis in China was −1.11 (95% confidence interval [CI]: −1.58 to −0.65) (Figure 2A). Except for the period 1992 to 1994, The APC for endometriosis-related primary infertility in China from 1990 to 2019 generally showed a downward trend. The ASPR declined most rapidly from 2006 to 2009, with an APC of −7.25 (95% CI:10.78 to −3.58) (Supplementary Table 2; Figure 2A).

From 1990 to 2021, the AAPC of ASPR for secondary female infertility associated with endometriosis, in China was −1.42 (95% CI: −1.64 to −1.20) (Figure 2B). On the whole, the APC for endometriosis-related secondary infertility in China also showed a downward trend. The ASPR declined most rapidly from 2006 to 2009, with an APC of −7.35 (95% CI: −9.16 to −5.49) (Supplementary Table 3; Figure 2B).

### Age-period-cohort analysis

**Figure 3:**
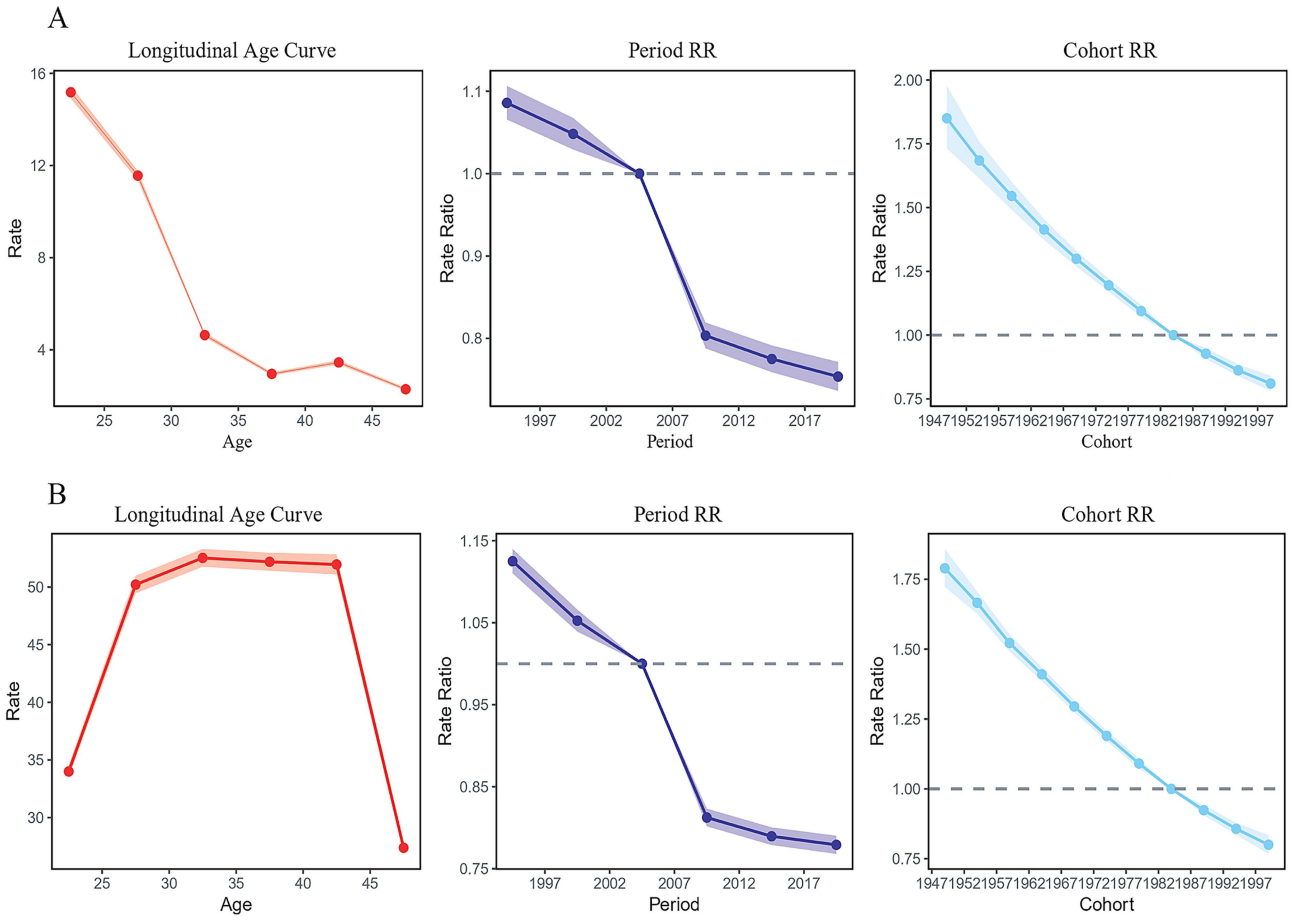
Age-period-cohort analysis for the prevalence of endometriosis-related infertility in China. **(A)** Primary infertility. **(B)** Secondary infertility.

The APC model was employed to gain deeper insights into shifts in the prevalence of endometriosis. The results of the APC model suggested that the prevalence rates of endometriosis-related primary infertility in China generally declined with increasing age when controlling for period and cohort effects. From the perspective of the period effect, taking the period 2002–2006 as the reference value (RR = 1), the prevalence rate of endometriosis-related primary infertility studies showed a monotonous decline throughout the study period. The prevalence rate diminished swiftly between 2002 and 2006 and 2007–2011, followed by a slower decline before and after these periods. In terms of the cohort effect, using the birth cohort from 1982 to 1991 as the reference value (RR = 1), we found that the age-standardized prevalence rate of endometriosis-related primary infertility declined with the successive birth cohort (Figure 3A).

Figure 3B indicates that when controlling for period and cohort effects, the prevalence rate of endometriosis-related secondary infertility in China is higher for individuals older than 25 years and rises with age while remaining stable between the ages of 25 and 40 years. Additionally, the prevalence of endometriosis-related female infertility in this age group is the highest in China, subsequently exhibiting a declining pattern as age progresses. From the perspective of the period effect, taking the 2002–2006 period group as the reference value (RR = 1), the prevalence of secondary infertility in women with endometriosis demonstrated a downward trajectory from 1990 to 2019, with the decreasing trend tending to slow after 2007–2011 period group. In terms of the cohort effect, using the birth cohort from 1982 to 1991 as the reference value (RR = 1), our analysis revealed that the age-standardized prevalence rate of secondary infertility in women with endometriosis-related diseases continued to decline over time (Figure 3B).

## Discussion

Most pelvic endometriosis includes three subtypes: superficial peritoneum, ovarian cysts and deep diseases, which are the main causes of infertility and pelvic pain, imposing a heavy burden on families and society (18). The underlying mechanisms of endometriosis-related infertility are still being investigated, and the condition is thought to be multifactorial (3). Endometriosis is a persistent inflammatory disease. Localized peritoneal inflammation sustains the expansion and persistence of endometriosis via endometrial-peritoneal adhesions, infiltration, neovascularization, cell multiplication, and tissue scarring. Furthermore, inflammatory processes contribute to the formation of pelvic adhesions and structural alterations, potentially impacting conception in individuals with endometriosis. These structural changes and physical factors may interfere with oocyte release from the ovaries, hinder ovum capture, or egg movement within the fallopian tube, and obstruct sperm transit into the fallopian tube (5). The distinctive proinflammatory characteristics of peritoneal fluid in patients with endometriosis are believed to compromise sperm functionality, resulting in sperm DNA fragmentation (19). Pain is an important clinical manifestation of endometriosis, and nearly all of the hormone treatments currently available are contraceptive pills, thus preventing conception (2). Given these complex pathophysiological interactions, the observed decline in endometriosis-related infertility prevalence in our study suggests significant advances in clinical management and public health interventions.

This study summarized the latest age-standardized prevalence of endometriosis-related female infertility in China and elucidated the effects of age, period, and cohort. This decline of endometriosis-related infertility may be attributed to several factors: (1) Enhanced healthcare access through China’s expanded medical insurance coverage for assisted reproductive technologies since 2022 (12, 20); (2) Improved endometriosis management protocols, including earlier laparoscopic interventions and optimized hormonal therapies (2, 21); (3) National policies promoting reproductive health education and preconception care. This finding suggests the need to control endometriosis-related female infertility. Nevertheless, this apparent reduction in disease burden might still contain some false-positive elements. Over the past three decades, diagnostic criteria for endometriosis have improved with advances in healthcare, and changes in these criteria may also affect the rate of endometriosis diagnosis.

The prevalence of endometriosis-related infertility varies by age group. The highest ASPR in China and the world for endometriosis-related primary infertility was in the 20–24 age group, while the highest ASPR for endometriosis-related secondary infertility in China was 40–44 age group, and the world was in the 35–39 age group. This trend may be influenced by practical factors. Most Chinese women marry before the age of 30 and hope to have their first child within this period. However, in recent years, the trend toward late childbearing has become increasingly obvious. From 2006 to 2017, the average age of first marriage for women rose from 23.6 years to 26.5 years, and the average age of first childbearing rose from 24.3 years to 27.3 years, both of which were delayed by 3 years. The rate of delay since 2012 was significantly larger than that in previous years, causing a superimposed effect on the declining fertility. The rate of delivery at tertiary hospitals for pregnant women over 35 years of age increased from 12.54% in 2015 to 17.43% in 2017 (22). Advanced maternal age is an important risk factor for infertility (22), which partially explains our results. Ovarian reserve declines significantly after age 35, with accelerated follicular atresia reducing oocyte quantity and quality (23). Concurrently, age-related mitochondrial dysfunction in oocytes increases aneuploidy rates, impairing embryo viability (24). According to the latest report on ART cycles conducted by the US Centers for Disease Control and Prevention in the United States in 2018, the live birth rate per startup cycle for women aged 41–42 was 13.2%, while for women under 35 it was 55.1% (25). These mechanisms, compounded by China’s trend toward delayed childbearing, collectively explain our observed age-specific burden patterns.

Li et al. observed a declining trend in the age-standardized incidence rate, prevalence, and years lived with disability (YLD) related to surgically confirmed endometriosis between 1990 and 2021 (26). Similarly, Chen et al. reported a global decrease in the burden of infertility attributable to endometriosis in 2021 (27). In recent years, a reduction has been noted in surgical and diagnostic procedures—particularly those concerning fibroids, female sterilization, and infertility treatment—thereby diminishing the likelihood of incidentally diagnosing endometriosis. These evolving clinical practices may have contributed to the decline in the incidence of surgically verified endometriosis (28). Oral contraceptives have been demonstrated to significantly reduce menstrual flow and may prevent the development of endometriosis by interfering with the implantation of retrograde endometrial cells (29). Furthermore, improvements in healthcare infrastructure and technological advances are considered potential factors underlying the decline in endometriosis-related primary infertility in China (30).

It is important to note that this decline in endometriosis-related infertility occurs against a backdrop of rising overall infertility rates in China, driven by broader societal trends such as delayed childbearing, increased educational attainment among women, and socioeconomic pressures (10, 22, 23, 31). This contrast highlights that the improvement in endometriosis management is a significant yet specific achievement within the larger, more complex challenge of declining fertility. In 2021, the global burden of endometriosis remained huge. Although the global burden of endometriosis has decreased from 1990 to 2021, there are still significant differences, especially in regions with low SDI (32).

The significant period effect observed—particularly the accelerated decline in endometriosis-related infertility prevalence after 2006—aligns with the pivotal developments in China’s healthcare landscape: The nationwide rollout of laparoscopic surgery for endometriosis diagnosis and treatment after 2005 enabling earlier intervention. These advances likely contributed to the steepest decline in ASPR during 2006–2009.

Our study has several limitations that should be considered when interpreting the results. First, the lack of provincial-level data impedes our exploration of regional heterogeneity and disparities in healthcare access across China’s vast territory. Second, the GBD data lack detailed clinical information, preventing us from accounting for important confounding factors such as the severity and subtype of endometriosis, individual treatment history, or key behavioral and environmental risk factors. Finally, while the APC model is a robust tool, it operates under certain statistical assumptions that may not fully capture the complex interactions between age, period, and cohort effects. Despite these limitations, our study provides a valuable national-level overview of long-term trends in endometriosis-related infertility in China.

## Conclusion

The ASPR of endometriosis-related female infertility in China and globally declined between 1990 and 2021. The decline in endometriosis-related primary infertility in China is lower than the overall global rate, whereas endometriosis-related secondary infertility in China has declined at a higher rate than the global average. This indicates that China has achieved specific improvements in the prevention and treatment of endometriosis-related female infertility. However, the general burden of endometriosis-related female infertility remains significant and needs continued attention.

## Data Availability

The datasets presented in this study can be found in online repositories. The names of the repository/repositories and accession number(s) can be found in the article/Supplementary material.

## Footnotes

^1^ https://vizhub.healthdata.org/gbd-results/
