# Supplementary material for: Endometriosis-related infertility in China: analysis of the global burden of disease study 2021
Source: Front Public Health. 2025 Oct 7;13:1651254. doi: 10.3389/fpubh.2025.1651254 (PMC12537762; doi:10.3389/fpubh.2025.1651254)
Supplement: Supplementary file 1 [file Table_1.docx]

Supplementary Tables

Supplementary Table 1. Global burden of endometriosis-related infertility in 1990, 2021, and the percentage change in age-standardized rates from 1990 to 2021.

Supplementary Table 2. Joinpoint regression analysis: Age-standardized prevalence and average annual percentage change of endometriosis-related primary infertility in China from 1990 to 2021.

Supplementary Table 3. Joinpoint regression analysis: Age-standardized prevalence and average annual percentage change of endometriosis-related secondary infertility in China from 1990 to 2021.

Supplementary Table 1. Global burden of endometriosis-related infertility in 2021 and the percentage change in age-standardized rates from 1990 to 2021.

|  |  |  | **1990(95% UI)** | | **2021(95% UI)** | | **（95% UI)** |
| --- | --- | --- | --- | --- | --- | --- | --- |
| **Location** | **Age** | **Cause** | **prevalence rate** | **Prevalence number** | **prevalence rate** | **Prevalence number** | **Percentage change in 1990-2021(Per 100,000)** |
| Global | 20-24 years | Primary infertility | 44.53 (17.78to90.84) | 108703.59 (43410.45to221769.49) | 31.09 (10.62to67.36) | 91320.39 (31188.42to197859.32) | -30.18% (-42.59%to-23.14%) |
| Global | 25-29 years | Primary infertility | 42.51 (15.79to95.53) | 93556.47 (34753.50to210252.44) | 30.1 (9.4to70.99) | 87598.43 (27352.49to206557.75) | -29.18% (-42.42%to-22.62%) |
| Global | 30-34 years | Primary infertility | 23.08 (7.74to54.51) | 43884.83 (14723.09to103629.44) | 15.49 (3.83to39.25) | 46304.41 (11435.67to117331.68) | -32.9% (-50.19%to-24.37%) |
| Global | 35-39 years | Primary infertility | 15.08 (4.82to37.14) | 26149.95 (8364.71to64426.20) | 11.04 (2.9to29.42) | 30665.09 (8054.9to81716.82) | -26.78% (-42.21%to-17.85%) |
| Global | 40-44 years | Primary infertility | 16.28 (4.76to37.11) | 22831.67 (6676.93to52032.85) | 12.48 (3.15to29.16) | 30951.12 (7826.25to72348.03) | -23.38% (-36.35%to-16.64%) |
| Global | 45-49 years | Primary infertility | 6.15 (1.22to16.36) | 7003.72 (1393.20to18619.60) | 4.55 (0.81to12.2) | 10713.59 (1914.61to28757.47) | -26.13% (-42.21%to-15.12%) |
| Global | All ages | Primary infertility | 12.15 (6.37to22.06) | 321684.98 (168707.31to584220.71) | 7.94 (3.95to14.9) | 312363.25 (155324.38to585825.54) | -34.61% (-41.35%to-28.54%) |
| Global | Age-standardized | Primary infertility | 11.39 (5.97to20.86) |  | 8.02 (3.97to15.18) |  | -29.61% (-37.19%to-24.79%) |
| China | 20-24 years | Primary infertility | 16.23 (3.27to45.21) | 10464.10 (2105.69to29153.71) | 12.3 (2.76to33.37) | 4219.89 (946.41to11449.8) | -24.2% (-34.82%to-4.52%) |
| China | 25-29 years | Primary infertility | 12.08 (2.12to37.81) | 6457.35 (1133.59to20211.67) | 10.08 (2.05to29.2) | 4121.08 (838.37to11931.88) | -16.51% (-28.13%to7.51%) |
| China | 30-34 years | Primary infertility | 5.18 (0.81to18.86) | 2185.10 (340.65to7961.93) | 4.32 (0.72to13.85) | 2526.95 (422.39to8098.54) | -16.5% (-31.67%to5.84%) |
| China | 35-39 years | Primary infertility | 3.40 (0.82to13.68) | 1762.46 (362.19to6032.47) | 3.01 (0.6to9.97) | 1551.74 (310.83to5142.77) | -24.73% (-37.95%to-8.51%) |
| China | 40-44 years | Primary infertility | 5.65 (1.04to17.56) | 1802.66 (332.02to5603.57) | 4.02 (0.81to12.05) | 1793.31 (361.57to5374.88) | -28.83% (-39.89%to-12.99%) |
| China | 45-49 years | Primary infertility | 3.89 (0.54to11.05) | 949.03 (131.69to2694.00) | 2.83 (0.35to8.2) | 1536.76 (189.33to4450.52) | -27.24% (-43.35%to-3.08%) |
| China | All ages | Primary infertility | 4.49 (1.63to10.44) | 25592.01 (9287.01to5949.03) | 2.36 (1.01to4.81) | 16401.43 (7009.5to33397.68) | -47.45% (-60.22%to-27.42%) |
| China | Age-standardized | Primary infertility | 3.66 (1.40to8.66) |  | 2.81 (1.16to6.16) |  | -23.35% (-32.68%to-10.52%) |
| Global | 20-24 years | Secondary infertility | 46.67 (17.79to94.99) | 116383.40 (43422.20to231901.91) | 40.86 (15.27to80) | 120025.44 (44842.71to234993.76) | -14.29% (-20.47%to-7.08%) |
| Global | 25-29 years | Secondary infertility | 75.50 (33.62to135.84) | 166184.87 (74004.25to298992.21) | 59.51 (25.86to106.75) | 173171.08 (75245.76to310621.49) | -21.18% (-26.15%to-16.92%) |
| Global | 30-34 years | Secondary infertility | 83.73 (41.21to146.97) | 159177.76 (78335.09to279401.05) | 61.66 (30.56to107.68) | 184329.24 (91346.69to321887.67) | -26.35% (-30.33%to-22.58%) |
| Global | 35-39 years | Secondary infertility | 87.39 (47.18to144.28) | 151575.76 (81838.28to250259.31) | 62.37 (33.78to103.3) | 173256.63 (93853.15to286981.53) | -28.63% (-33.26%to-25.14%) |
| Global | 40-44 years | Secondary infertility | 87.96 (44.47to150.49) | 123346.28 (62352.83to211021.67) | 61.66 (31.78to104.37) | 152971.34 (78854.87to258943.05) | -29.9% (-33.86%to-26.5%) |
| Global | 45-49 years | Secondary infertility | 29.60 (6.01to77.54) | 33681.08 (6843.91to88245.45) | 21.45 (3.96to58.34) | 50540.71 (9333.58to137472.91) | -27.53% (-40.19%to-15.21%) |
| Global | All ages | Secondary infertility | 29.03 (16.49to47.66) | 768768.85 (436673.23to1261864.44) | 22.22 (12.84to36.75) | 873542.61 (504746.57to1445036.48) | -23.48% (-26.83%to-19.97%) |
| Global | Age-standardized | Secondary infertility | 29.18 (16.68to47.92) |  | 21.98 (12.7to36.31) |  | -24.7% (-27.99%to-21.11%) |
| China | 20-24 years | Secondary infertility | 47.01 (16.92to89.58) | 30314.85 (10910.32to57767.51) | 27.77 (10.16to54.42) | 9526.98 (3484.06to18670.25) | -40.93% (-51.01%to-31.7%) |
| China | 25-29 years | Secondary infertility | 74.51 (38.65to126.69) | 39837.76 (20665.69to67733.10) | 44.11 (21.9to74.71) | 18026.64 (8948.68to30532.92) | -40.8% (-48.62%to-33.09%) |
| China | 30-34 years | Secondary infertility | 81.63 (41.85to141.25) | 34466.49 (17670.31to59635.93) | 49.81 (26.24to84.33) | 29123.42 (15341.92to49305.11) | -38.99% (-45.71%to-30.78%) |
| China | 35-39 years | Secondary infertility | 86.25 (46.86to144.67) | 38047.22 (20670.69to63816.37) | 53.75 (29.97to85.75) | 27734 (15461.91to44244.95) | -37.68% (-44.3%to-29.95%) |
| China | 40-44 years | Secondary infertility | 91.46 (46.15to156.22) | 29189.19 (14727.04to49857.34) | 58.7 (31.43to99.04) | 26185.78 (14019.79to44181.35) | -35.82% (-41.74%to-29.21%) |
| China | 45-49 years | Secondary infertility | 45.63 (6.86to114.75) | 11124.88 (1673.64to27976.00) | 31.82 (5.37to78.73) | 17263.25 (2914.22to42720.08) | -30.28% (-46.36%to-2.41%) |
| China | All ages | Secondary infertility | 32.88 (18.78to52.46) | 187284.94 (106950.83to298840.49) | 18.59 (11.09to29.98) | 129108.81 (77065.6to208229.95) | -43.47% (-51.01%to-34.84%) |
| China | Age-standardized | Secondary infertility | 29.91 (16.94to48.26) |  | 18.49 (11.05to29.31) |  | -38.16% (-43.81%to-31.53%) |

Supplementary Table 2. Joinpoint regression analysis: Age-standardized prevalence and average annual percentage change of endometriosis-related primary infertility in China from 1990 to 2021.

| **Measure** | **Age** | **Location** | **Cause** | **Segment.Start**  **(Year)** | **Segment.End**  **(Year)** | ***p*** | **APC**  **95% CI** |
| --- | --- | --- | --- | --- | --- | --- | --- |
| Prevalence | Age-standardized | China | Endometriosis-related primary infertility | 1992 | 1994 | <0.05 | 2.81 (-1.1 to 6.87) |
| Prevalence | Age-standardized | China | Endometriosis-related primary infertility | 1994 | 2006 | <0.05 | -1.2 (-1.45 to -0.94) |
| Prevalence | Age-standardized | China | Endometriosis-related primary infertility | 2006 | 2009 | <0.05 | -7.25 (-10.78 to -3.58) |
| Prevalence | Age-standardized | China | Endometriosis-related primary infertility | 2009 | 2021 | <0.05 | -0.08 (-0.31 to 0.15) |

Supplementary Table 3. Joinpoint regression analysis: Age-standardized prevalence and average annual percentage change of endometriosis-related secondary infertility in China from 1990 to 2021.

| **Measure** | **Age** | **Location** | **Cause** | **Segment.Start**  **(Year)** | **Segment.End**  **(Year)** | ***p*** | **APC**  **95% CI** |
| --- | --- | --- | --- | --- | --- | --- | --- |
| Prevalence | Age-standardized | China | Endometriosis-related secondary infertility | 1992 | 2006 | <0.05 | -1.23 (-1.32 to -1.14) |
| Prevalence | Age-standardized | China | Endometriosis-related secondary infertility | 2006 | 2009 | <0.05 | -7.35 (-9.16 to -5.49) |
| Prevalence | Age-standardized | China | Endometriosis-related secondary infertility | 2009 | 2017 | <0.05 | 0.38 (0.12 to 0.65) |
| Prevalence | Age-standardized | China | Endometriosis-related secondary infertility | 2017 | 2021 | <0.05 | -1.09 (-1.71 to -0.47) |
